# ‘*Candidatus* Liberibacter Asiaticus’ SDE1 Effector Induces Huanglongbing Chlorosis by Downregulating Host *DDX3* Gene

**DOI:** 10.3390/ijms21217996

**Published:** 2020-10-27

**Authors:** Yinghui Zhou, Xiangying Wei, Yanjiao Li, Zhiqin Liu, Yongping Duan, Huasong Zou

**Affiliations:** 1 State Key Laboratory of Ecological Pest Control for Fujian and Taiwan Crops, College of Plant Protection, Fujian Agriculture and Forestry University, Fuzhou 350002, China; zyh7426@163.com (Y.Z.); liyanjiao0810@163.com (Y.L.); lzqfujian@126.com (Z.L.); 2 Institute of Oceanography, Minjiang University, Fuzhou 350108, China; xiangyingwei@mju.edu.cn; 3 U.S. Horticultural Research Laboratory, Agricultural Research Service, United States Department of Agriculture, Fort Pierce, FL 34945, USA; yongping.duan@ars.usda.gov

**Keywords:** *Candidatus* Liberibacter asiaticus, SDE1, NbDDX3, chlorosis, CsDDX3

## Abstract

‘*Candidatus* Liberibacter asiaticus’ (CLas) is the pathogenic bacterium that causes the disease Huanglongbing (HLB) in citrus and some model plants, such as *Nicotiana benthamiana*. After infection, CLas releases a set of effectors to modulate host responses. One of these critical effectors is Sec-delivered effector 1 (SDE1), which induces chlorosis and cell death in *N. benthamiana*. In this study, we revealed the DEAD-box RNA helicase (DDX3) interacts with SDE1. Gene silencing study revealed that knockdown of the *NbDDX3* gene triggers leaf chlorosis, mimicking the primary symptom of CLas infection in *N. benthamiana*. The interactions between SDE1 and NbDDX3 were localized in the cell membrane. Overexpression of *SDE1* resulted in suppression of *NbDDX3* gene expression in *N. benthamiana*, which suggests a critical role of *SDE1* in modulating *NbDDX3* expression. Furthermore, we verified the interaction of SDE1 with citrus DDX3 (CsDDX3), and demonstrated that the expression of the *CsDDX3* gene was significantly reduced in HLB-affected yellowing and mottled leaves of citrus. Thus, we provide molecular evidence that the downregulation of the host *DDX3* gene is a crucial mechanism of leaf chlorosis in HLB-affected plants. The identification of CsDDX3 as a critical target of SDE1 and its association with HLB symptom development indicates that the *DDX3* gene is an important target for gene editing, to interrupt the interaction between DDX3 and SDE1, and therefore interfere host susceptibility.

## 1. Introduction

Citrus Huanglongbing (HLB) is one of the most destructive citrus diseases causing severe losses in citrus production [1]. The disease is associated with three species of phloem-limited pathogenic liberibacters, which include *Candidatus* Liberibacter asiaticus (CLas), *Candidatus* Liberibacter africanus, and *Candidatus* Liberibacter americanus [2]. Among the three species, the CLas pathogen is the most prevalent and is vectored by the Asian citrus psyllid (*Diaphorina citri*), thus naturally spreading the pathogen among citrus plants [1]. The liberibacters can also be transmitted by dodder (*Cuscuta*) and through grafting. Besides, HLB affects other plant species, such as *Catharanthus roseus*, *Solanum lycopersicum*, *Nicotiana tabacum*, and *N. benthamiana* [3,4,5,6]. The characteristic disease symptoms include yellow shoot and formation of blotchy mottles on leaves. In addition, the fruits sometimes exhibit uneven discoloration with aborted seeds and altered ripening dynamics [7].

CLas-infected citrus plants exhibit a wide variety of physiological and biochemical disorders. Within three months to one year after infection, CLas bacteria are disseminated to all tissues and organs [8]. Along with detection of CLas replication in sieve tubes, HLB can be diagnosed by a high accumulation of starches, callose deposition, and formation of multiple necrotic pockets [9,10]. Since the expression of photosynthetic genes is down-regulated in HLB, photoassimilated export in the leaves is impaired [11,12]. As a result, the leaves exhibit chlorosis [13,14].

Ca. Liberibacter asiaticus secretes a minimum of 86 proteins via the general secretion system (Sec pathway) [15]. Many of these Sec-delivered effectors (SDEs) have different expression levels in citrus and psyllids to manipulate host responses [16,17,18]. Sec-delivered effector 1 (SDE1, also known as Las5315) is a protein composed of 154 amino acids and can be secreted to the extracellular space outside of the cell through the Sec pathway, upon expression in *Escherichia coli* [15]. The transient expression of mature SDE1 without a signal peptide in *Agrobacterium*-mediated transformed *N. benthamiana* plants induces cell death. On the contrary, full-length *SDE1* cannot induce cell death [6]. In addition, the deletion of a chloroplast targeting sequence results in starch accumulation and leaf chlorosis, but not cell death [6]. These findings indicate that SDE1 plays a pleiotropic role in its diverse interactions with host proteins. The first characterized SDE-interacting protein in citrus was papain-like cysteine proteases. However, the role(s) of these interactions in HLB progression remains unclear [19].

The objective of this study was to identify the molecular mechanisms underlying *SDE1*-mediated host plant responses. We found that host SDE1 targets DEAD-box RNA helicase DDX3, and that downregulation of host *DDX3* gene is associated with leaf chlorosis. The findings of the present study increase our understanding of CLas pathogenesis and HLB symptom development.

## 2. Results

### 2.1. The Full-Length SDE1 Gene Is Not Transcribed during Agrobacterium-Mediated Transient Expression in N. benthamiana

**Figure 1:**
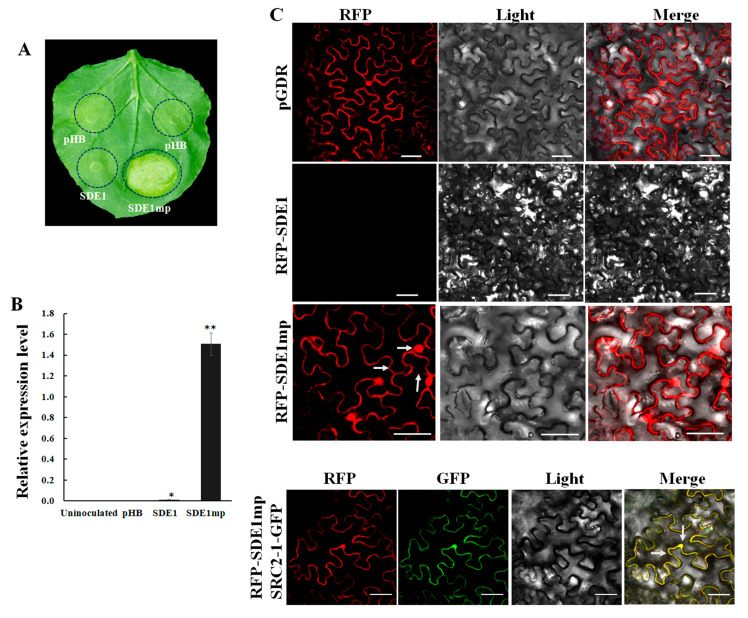
Expression of *SDE1* in *N. benthamiana* by agroinfiltration. (**A**) Chlorosis induced by SDE1mp. The phenotype was observed at 3 dpi. (**B**) qRT-PCR assay of the transcript levels of *SDE1* and *SDE1mp* at 3 dpi. The relative expression levels were normalized to internal control *NbEF1a*. Asterisks indicate significant differences (* *p* < 0.05, ** *p* < 0.01, *n* = 3). (**C**) Visualization of RFP-SDE1 and RFP-SDE1mp fusion proteins in *N. benthamiana* cells. Arrows indicate the localization of RFP-SDE1mp in the cell membrane, cytoplasm, and nucleus. Samples were examined under the microscope at 2 dpi. Bar denotes 50 μm. All experiments were repeated three times.

The expression levels of *SDE1* were measured in the leaves of *Agrobacterium*-transformed *N. benthamiana* plants to understand why induced cell death did not occur after transient expression of the full-length *SDE1* [20]. The coding sequences of full-length SDE1 and mature SDE1 (SDE1mp) were separately cloned into the binary vector pHB (Figure S1). While the transient expression of *SDE1mp* induced chlorosis in *N. benthamiana* at 3 days post infiltration (dpi), no chlorosis was observed in the infiltration area after transient expression of the full-length *SDE1* (Figure 1A). The results of quantitative RT-PCR (qRT-PCR) showed a high transcript level of *SDE1mp*. In contrast, the transcript level of *SDE1* was extremely low (Figure 1B). In all qRT-PCR analyses, no *SDE1* transcripts were detected from uninoculated leaves, or the leaves infiltrated with *Agrobacterium* harboring the empty vector pHB (Figure 1B). To evaluate the expression of the SDE1 protein, SDE1 and SDE1mp were fused to the C-terminus of a red fluorescent protein (RFP) in a pGDR vector. At 2 dpi, *N. benthamiana* cells expressing the RFP-SDE1mp fusion emitted a strong fluorescence signal, and the signal was distributed in the cell membrane, cytoplasm, and nucleus (Figure 1C). In contrast, cells not expressing the RFP-SDE1 fusion did not emit any fluorescence (Figure 1C). RFP-SDE1mp was additionally co-expressed with SRC2-1-GFP which is expressed in the membrane and nucleus. In this case, yellow fluorescence was observed from the cell membrane and nucleus (Figure 1C). The results demonstrated that the full-length *SDE1* did not induce cell death in *N. benthamiana,* because it could not be transcribed during *Agrobacterium*-mediated transient expression. Consequently, the mature protein SDE1mp was selected to test *N. benthamiana* phenotypes in our subsequent experiments.

### 2.2. SDE1 Interacts with the N. benthamiana DDX3 

**Figure 2:**
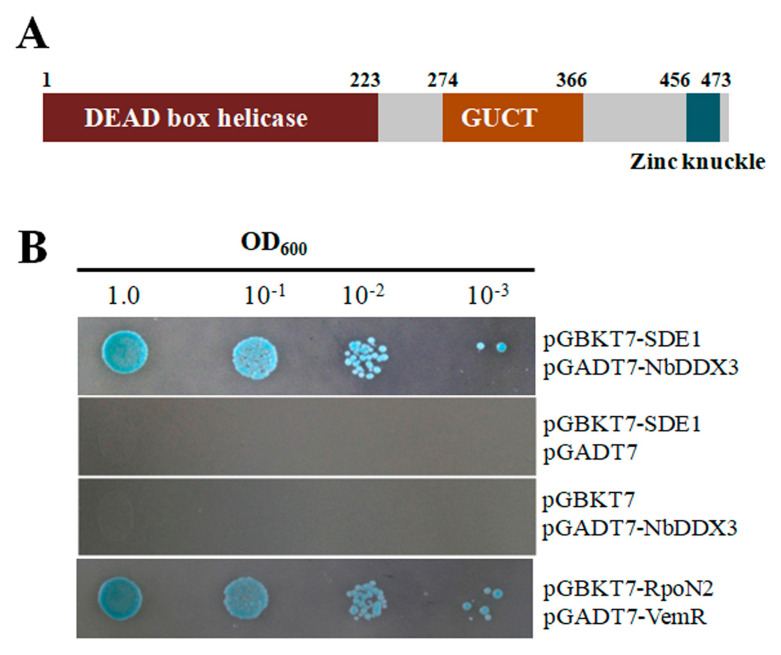
Screening and characterization of NbDDX3 that interacted with SDE1 from *N. benthamiana.* (**A**) NbDDX3 domain structures encoded by cDNA harbored in pGADT7. Different color boxes indicate the locations of DEAD-box helicase, RNA-binding GUCT, and zinc knuckle. (**B**) AH109 cells transformed with pGADT7 plasmids and pGBKT7-SDE1 grown on SD/-Ade/-Leu/-Trp/-His media supplemented with 20 μg/mL X-α- galactosidase. The interaction between VemR and RpoN2 was used as a positive control. Y2H assays were repeated three times.

Using SDE1 as bait, SDE1-interacting proteins were screened from a *N. benthamiana* cDNA library. The pGADT7 plasmids were recovered from yeast for DNA sequencing, and two duplicate clones were identified. The cDNA had a 133-bp untranslated region (UTR) at the 5’ terminus and a 1413-bp open reading frame (ORF) encoding a DEAD-box RNA helicase of the *DDX3* gene. The predicted product was a 477 amino acid protein. The N-terminal amino acids 1 to 219 code for an ATP-dependent RNA helicase domain, amino acid positions 274 to 366 are RNA-binding GUCT domain, while amino acid positions 456 to 473 form a zinc knuckle domain (Figure 2A). The recovered pGADT7 vector and pGBKT7-SDE1 were co-transformed into AH109 cells to repeat yeast two-hybrid (Y2H) performances. Interaction between SDE1 and NbDDX3 was determined by evaluating the β-galactosidase activity of the transformant. As shown in Figure 2B, SDE1 was interacted with NbDDX3.

### 2.3. Interaction between SDE1 and NbDDX3 Is Localized at the Cell Membrane

**Figure 3:**
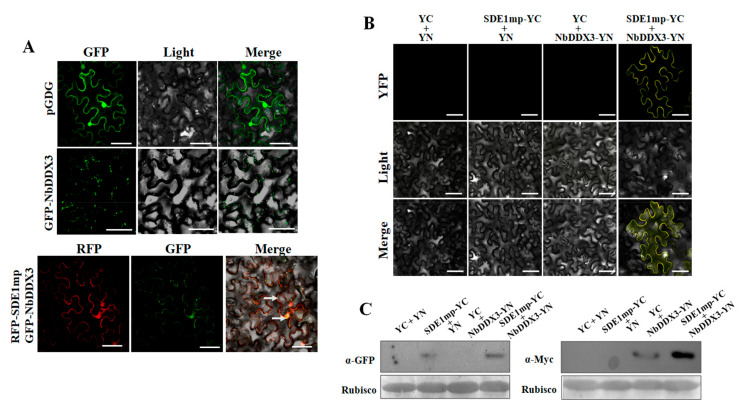
Subcellular localization of NbDDX3 and its interaction with SDE1mp in *N. benthamiana* cells. (**A**) Subcellular localization and co-localization of RFP-SDE1mp and GFP-NbDDX3 on the cell membrane at 2 dpi. (**B**) Bimolecular fluorescence complementation analysis showing the spatial interaction of SDE1mp and NbDDX3. The bar represents 50 μm. (**C**) Infiltrated plant leaves were collected for immunoblotting assays with anti-GFP and anti-Myc antibodies for evaluation of protein expression levels. Ponceau S staining indicates equal loading of proteins. Samples were examined under the microscope at 2 dpi. All experiments were repeated three times.

A GFP-NbDDX3 fusion was constructed to investigate its in vivo interaction with SDE1mp. Fluorescent signal detection revealed that GFP-NbDDX3 accumulated to high levels in cytoplasmic vesicles, with small quantities localized in the cell membrane (Figure 3A). To examine the spatial interaction, GFP-NbDDX3 and RFP-SDE1mp were co-expressed in *N. benthamiana* cells. RFP-SDE1mp was observed in the cell membrane, nucleus, and cytoplasm. Nevertheless, the subcellular location of GFP-NbDDX3 was altered. A portion of NbDDX3 was located in the nucleus, but not in the cell membrane and organelles. Furthermore, additional NbDDX3 proteins were recruited to the cell membrane. GFP-NbDDX3 and RFP-SDE1mp were co-localized at the cell membrane and nucleus (Figure 3A). A bimolecular fluorescence complementation assay was performed to further confirm the interaction between NbDDX3 and SDE1mp. Results showed the emission of a clear yellow fluorescence from the cell membranes (Figure 3B). In addition, the expression levels of SDE1mp-YC and NbDDX3-YN were evaluated by western blot analysis (Figure 3C), and the results suggested that a fraction of NbDDX3 interacted with SDE1mp at the cell membrane.

### 2.4. Silencing of NbDDX3 Results in Mottled Leaves with Chlorosis

**Figure 4:**
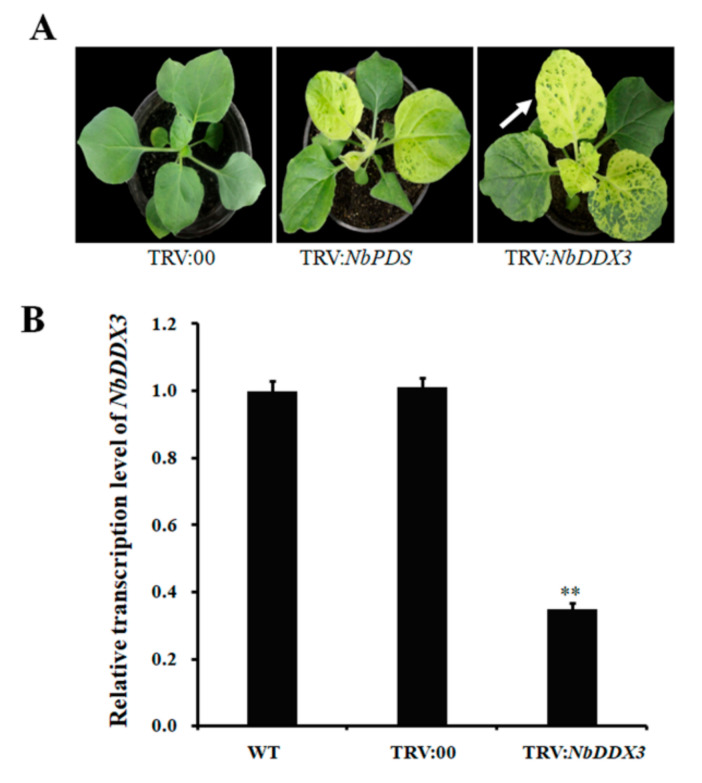
Silencing of the *N. benthamiana NbDDX3* gene. (**A**) Growth phenotypes of *NbDDX3-*silenced plants at 15 dpi. White arrows indicate leaf mottling in NbDDX3-silenced plant. (**B**) qRT-PCR analysis of the transcript level of the *NbDDX3* gene at 15 dpi. The expression of *NbEF1α* was used as an internal control. The expression level in wild type plants was set to “1” and the fold change in silenced plants was calculated by comparison with wild type. Error bars indicate standard deviations, and asterisks denote statistical significance (** *p* ˂ 0.01, *n* = 3). All experiments were repeated three times.

To determine the function of the *NbDDX3* gene, we generated plants in which the respective transcripts were knocked down by virus-induced gene silencing (VIGS) using the tobacco rattle virus (TRV) vector. At 15 dpi, *NbDDX3-*silenced plants exhibited yellow coloration on the leaves, which was similar to the silencing of the phytoene desaturase gene (*PDS*) in positive control plants (Figure 4A). The mottled phenotype in *NbDDX3-*silenced plants was observed on the first three leaves after inoculation with a TRV construct. Meanwhile, late budding leaves were completely bleached upon maturation. The silencing efficiency of the *NbDDX3* gene was evaluated in mottled leaves by qRT-PCR analysis. Relative to the wild type and the TRV:00 control, the transcript level of *NbDDX3* was reduced by 65% in gene silenced plants (Figure 4B).

### 2.5. Transient Expression of SDE1 Suppresses NbDDX3 Gene Expression

**Figure 5:**
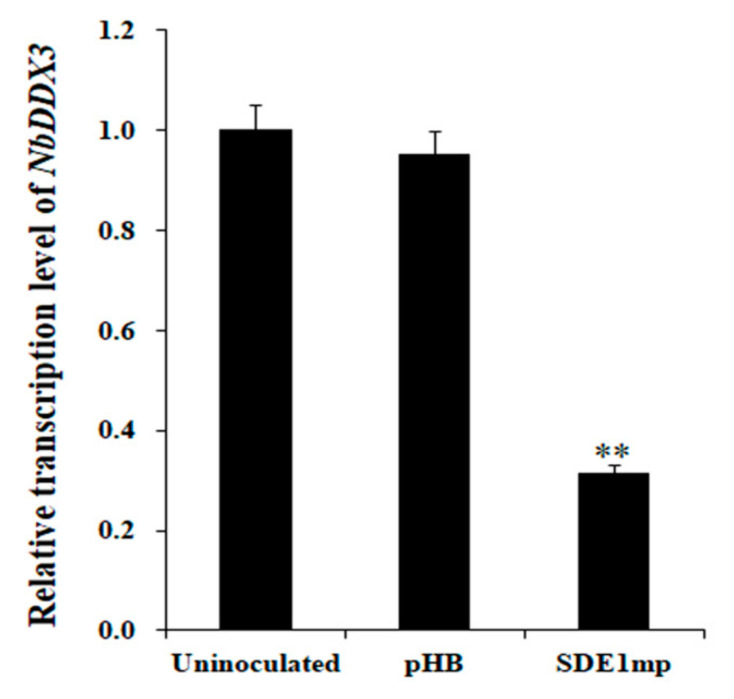
*NbDDX3* transcript levels in *N. benthamiana* plants transiently expressing *SDE1mp*. qRT-PCR analysis was performed at 2 dpi. The expression of *NbEF1a* was used as an internal control. The level of expression of *NbDDX3* in uninoculated plants was set to “1”. The fold change in plants expressing *SDE1mp* was calculated by comparison with uninoculated plants. Error bars indicate standard deviations, and asterisks denote statistical significance (** *p* ˂ 0.01, *n* = 3).

The yellow coloration phenotype on the leaves of *NbDDX3*-silenced plants prompted us to know the transcription pattern of *NbDDX3* in response to the expression of *SDE1*. The transcript levels of *NbDDX3* in *N. benthamiana* leaves transiently expressing the *SDE1mp* gene were determined. Relative to the uninoculated control at 2 dpi, the expression level of *NbDDX3* was reduced by 70% in plants expressing *SDE1mp* (Figure 5). This result suggests that transient expression of the *SDE1mp* gene in *N. benthamiana* significantly suppressed transcription of the *NbDDX3* gene. Further, this result indicates that the chlorosis induced by *SDE1mp* was caused by the down-regulation of *NbDDX3* transcription.

### 2.6. The Citrus Homolog CsDDX3 Interacts with SDE1 and Shows Reduced Expression in HLB-Infected Leaf Tissue

**Figure 6:**
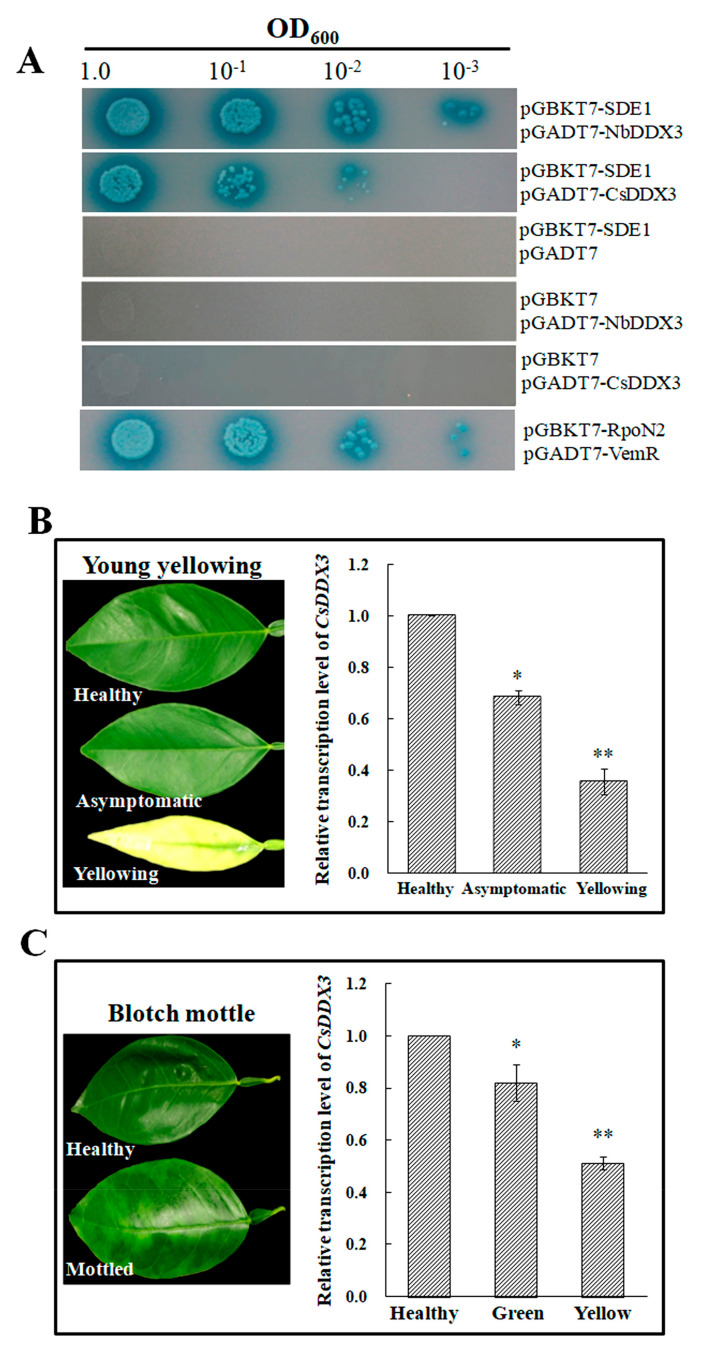
The association of *CsDDX3* with *SDE1* and yellowing HLB-infected citrus leaves. (**A**) Y2H showing interaction of CsDDX3 with SDE1. AH109 transformants were grown on SD/-Ade/-Leu/-Trp/-His media supplemented with 20 μg/mL X-α- galactosidase. (**B**,**C**) Relative transcription level of *CsDDX3* in young yellowing and mature blotch-mottled citrus leaves. The leaves show representative examined samples. The expression of *CsCOX* was used as an internal control. The expression level of *CsDDX3* in healthy plants was set to “1”. The fold change in other samples was calculated by comparison with healthy leaves. Error bars indicate standard deviations, and asterisks denote statistical significance (* *p* < 0.05, ** *p* ˂ 0.01, *n* = 3). All experiments were repeated three times.

The homolog CsDDX3 was cloned from grapefruit (*Citrus paradise* Macf. cv. *Duncan*) to examine the interaction with SDE1. The *CsDDX3* gene is a 1488-bp long transcript that encodes a 495 amino acid protein. The amino acids share 73% identity with NbDDX3. Besides, the RNA helicase and RNA-binding GUCT domains of NbDDX3 and CsDDX3 are highly conserved. The most varied region is the C-terminus from amino acid 374, but the C-terminal zinc knuckle (-GACFSCGRSGHRASECPN-) is conserved (Figure S2). Similar to NbDDX3, the CsDDX3 interacts with effector SDE1 (Figure 6A). The transcript levels of the *CsDDX3* gene were studied in HLB-infected citrus leaves. In young leaves, the transcript level of *CsDDX3* was reduced by 33% in asymptomatic leaves lacking the yellowing phenotype and reduced by 67% in yellowing leaves, relative to healthy leaves (Figure 6B). In mature leaves showing mottled symptoms, the transcript levels of *CsDDX3* in green sections were reduced by 20% and reduced by 50% in the yellow areas, relative to healthy leaves (Figure 6C).

## 3. Discussion

In this study, we revealed that NbDDX3 is an SDE1-interacting protein, and demonstrated that the *SDE1*-induced downregulation of *NbDDX3* is the cause of chlorosis. Furthermore, we confirmed that SDE1 also interacts with the CsDDX3 in the natural host, citrus. In HLB-infected citrus leaves, the transcriptional expression of *CsDDX3* is down-regulated, especially in yellowing tissues. These results provide solid evidence to illustrate that specific molecular events are associated with the chlorosis phenotype observed in HLB.

The structural components of SDE1 were deciphered by bioinformatic analysis. The N-terminal 24 amino acids represent a signal peptide, which is presumably cleaved by CLas upon translocation [6]. *Agrobacterium*-mediated transient expression of the mature SDE1 without a signal peptide (SDE1mp) induces cell death and chlorosis in *N. benthamiana*. However, the full-length *SDE1* does not induce any HLB related phenotype [6]. To further understand this difference, the expression of *SDE1* was examined at mRNA transcriptional and protein translational levels. Our results showed that following *Agrobacterium*-mediated transient expression assays, the full-length *SDE1* was not transcribed. How the signal peptide coding sequence of the full-length SDE1 affects the 35S promoter in the expression cassette remains to be elucidated.

A previous study reported that SDE1 is localized to the chloroplast vesicles and induces cell death in *N. benthamiana* [20]. With the exception of the signal peptide at the N-terminus, a chloroplast targeting sequence was found from a neighboring SDE1 signal peptide. The deletion of the sequence switches cell death signals to the chlorotic phenotype [6]. Using a different fusion system, this study identified several subcellular localizations for RFP-SDE1mp, including the cell membrane, protoplasm, and nuclei. In addition to the *NbDDX3* involved in leaf chlorosis, a 26S proteasome non-ATPase regulatory subunit (PSMD14) Niben101Scf07364g00017.1, an ARM repeat protein Niben101Scf05290g02006.1, and Niben101Scf04231g02014.1 were screened from the cDNA library (data not shown). The silencing of *NbPSMD14* triggered leaf death (Figure S3). These results support indicate that *SDE1* may target several host genes to manipulate diverse plant responses.

DDX3 is involved in various stages of gene expression, such as transcription, mRNA maturation, mRNA export, and translation [21]. Although the role of DDX3 in citrus has not been fully elucidated, studies on the DEAD-box RNA helicases of other plants provide solid evidence regarding their roles in growth, development, and response to biotic and abiotic stresses [22,23,24]. Furthermore, DEAD-box RNA helicases are not limited to chloroplasts, albeit they were first identified as chloroplast-localized protein. For example, the *Arabidopsis* RNA helicase LOS4 is localized in the cytoplasm and enriched at the nuclear rim [22]. Besides, two stress response suppressors, STRS1 and STRS2, are localized in the nucleolus and nucleoplasm [23]. This study showed that the *N. benthamiana* NbDDX3 is localized to the cell membrane and in cytoplasmic vesicles. Co-expression of *NbDDX3* and *SDE1* triggered the recruitment of NbDDX3 proteins to the cell membrane, facilitating its interaction with SDE1.

Considering that silencing *NbDDX3* resulted in leaf chlorosis, we hypothesized a downregulation of host *DDX3* genes would be involved in the chlorotic phenotype. The expression of *SDE1mp* in *N. benthamiana* did exert a suppression effect on *NbDDX3* transcription. Thereafter, the expression of the *CsDDX3* gene was further studied in HLB-affected citrus leaves. In comparison with healthy plants, *CsDDX3* was expressed at low transcription levels in mottled and yellowing leaf tissues. Although the mechanism by which *SDE1* affects the expression of *CsDDX3* is unknown, we showed that like NbDDX3, CsDDX3 can interact with SDE1. We concluded that the downregulation of *NbDDX3* and *CsDDX3* induces HLB chlorosis in host plants.

In conclusion, this study identified citrus CsDDX3 and tobacco NbDDX3 as SDE1-interacting proteins. Downregulation of either *CsDDX3* or *NbDDX3* genes was associated with leaf chlorosis. Our study provides new insights into the molecular basis underlying CLas pathogenesis and HLB symptom development. The identification of CsDDX3 as a critical target of *SDE1* and its association with HLB symptom indicates that it could be an intriguing gene editing target, to interrupt SDE1-CsDDX3 interactions and, therefore, manipulate the host susceptibility.

## 4. Materials and Methods

### 4.1. Plant and Bacterial Materials

*N. benthamiana* was grown in a greenhouse under long day conditions (16 hr light/8 hr dark) at Fujian Agriculture and Forestry University. *C. paradisi* plants were grown in a greenhouse at the U.S. Horticultural Research Laboratory of USDA. The plasmids and strains used in this study are listed in Table S1. *E. coli* and *A. tumefaciens* strains were cultured in Luria-Bertani media at 37 °C and 28 °C, respectively, while the yeast strain AH109 was cultured in YPD media (1% yeast extract, 2% peptone, and 2% glucose) at 30 °C. Antibiotics were used at the following concentrations: Kanamycin (Km), 50 μg/mL; rifampicin (Rif), 50 μg/mL; ampicillin (Ap), 50 μg/mL; spectinomycin (Sp), 25 μg/mL.

### 4.2. DNA Manipulation and Plasmid Construction

DNA isolation, restriction enzyme digestion, and plasmid transformation were performed using standard methods [25]. The PCR primers used for molecular cloning and qRT-PCR analysis are listed in Table S2.

To evaluate the transcription levels in agroinfiltration, the coding sequences of full-length *SDE1* and signal peptide-deleted *SDE1mp* were separately cloned onto the binary vector pHB at *Bam*H I and *Sac* I sites (Table S1). The coding sequence of *SDE1mp* was fused to the C-terminus of RFP in pGDR at *Sal* I and *Bam*H I sites to examine its subcellular localization and co-localization. NbDDX3 was fused to the C-terminus of GFP in pGDG. For bimolecular fluorescence complementation analysis, the coding sequences of *SDE1mp* and *NbDDX3* were cloned into 1301-YC and 1301-YN at *Xba* I and *Kpn* I sites, respectively (Table S1). 1301-YC and 1301-YN were split YFP vectors for detecting protein interaction in living cells [26]. To isolate the interacting protein from *N. benthamiana*, the coding sequence of the *SDE1* gene was cloned onto pGBKT7 at *Eco*R I and *Pst* I sites to generate pGBKT7-SDE1. The CTG alternative translation start codon of the *SDE1* gene was replaced by ATG in the forward primer (Table S2). Primers CsDDX3.F and CsDDX3.R were used to amplify the 1488-bp *CsDDX3* gene fragment, which was inserted into pGADT7 at *Eco*R I and *Pst* I sites. Then, the plasmids were transformed into *A. tumefaciens* strain GV3101 by electroporation.

### 4.3. Agrobacterium-Mediated Transient Expression

The *A. tumefaciens* strain GV3101 harboring corresponding constructs was cultivated overnight in LB media. The cells were harvested and suspended in a buffer (10 mM MgCl_2_, 10 mM MES, and 200 μM acetosyringone, pH 5.7) to a final concentration of OD_600_ = 0.2. After 2 h incubation, GV3101 cells were injected into the leaves of 4 weeks-od *N. benthamiana* grown in a growth room at 25 °C with a 14 h light/10 h dark cycle. *N. benthamiana* leaves were collected at 2 dpi and examined and imaged under a Leica confocal laser scanning microscope (SP8, Leica, Wetzlar, Germany) to detect the localization of the fluorescent fusion protein. Three different samples were examined under the microscope for each experiment. A SRC2-1-GFP construct was used for co-transformation with pGDR-SDE1mp to provide additional confirmation that the SDE1 protein is localized to cell membrane and nucleus [27]. The phenotypes of *N. benthamiana* leaves induced by *SDE1* and *SDE1mp* were observed at 3 dpi, and all the experiments were repeated four times.

### 4.4. qRT-PCR Analysis

RNA was extracted from *N. benthamiana* as previously described [28]. qRT-PCR experiments were performed on a CFX Connect real-time system (Bio-Rad, Hercules, CA, USA) using iTaq Universal SYBR Green Supermix (Bio-Rad, Shanghai, China). The PCR thermal cycle conditions were as follows: denaturation at 95 °C for 30 s and 40 cycles at 95 °C for 5 s; 58 °C for 20 s. The expression of *NbEF1a* was used as an internal control for gene expression analysis. The relative expression level was determined, and statistical analysis was performed using the CFX Maestro software (Bio-Rad). The average threshold cycle was normalized according to the internal control in the Mode of Normalized expression (∆∆Cq). Each experiment was repeated three times.

To evaluate the expression level of *CsDDX3* in citrus plants, asymptomatic, symptomatic yellow, and blotch mottle leaves were separately collected from *C. paradisi*. For blotch mottle leaves, yellow and green sectors of each leaf were separated. Total RNAs were extracted using the RNeasy Plant Mini Kit (Qiagen, Hilden, Germany). After quantification, qRT-PCR reactions were carried out to determine the expression level of *CsDDX3* in green and yellow sections of citrus leaves. The relative expression levels were normalized and calibrated according to the comparative CT (2^−ΔΔCT^) method using *CsCOX* as an internal control [29]. The relative expression level was expressed as the mean ± S.E of three replicates.

### 4.5. Yeast Two-Hybrid Assay

A yeast two-hybrid assay was performed to screen SDE1-interacting proteins from a cDNA library of *N. benthamiana*. The initial Y2H screening was performed on SD/-Leu/-Trp/-His/media using SDE1 as bait. Then, the pGADT7 vector positive clones were obtained for DNA sequencing and subsequent BLAST search. These plasmids were subsequently used to repeat the Y2H experiment to confirm interaction with SDE1. The positive clones were cultivated on SD/-Ade/-Leu/-Trp/-His/media supplemented with 20 μg/mL of X-α-galactosidase. The interaction between VemR and RpoN2 was used as a positive control [30]. Y2H experiment was performed three times.

The interaction between SDE1 and CsDDX3 was examined after co-transformation of yeast AH109 with pGBKT7-SDE1 and pGADT7-CsDDX3. This experiment was repeated three times.

### 4.6. Virus-Induced Gene Silencing

Virus-induced gene silencing was performed to study the function of *NbDDX3*. Partial sequences of the *NbDDX3* gene were PCR amplified, and the amplicons were inserted into a pTRV2 vector. The resultant pTRV2 constructs were transfected into *A. tumefaciens* strain GV3101 via electroporation. Then, GV3101 cultures containing a mixture of pTRV1 and pTRV2 (1:1, *v*/*v*) were co-infiltrated into 20-day-old *N. benthamiana* leaves. A mixture of pTRV1 and pTRV2 empty vectors was used as a negative control, while a combination of pTRV1 and TRV-PDS was used as a control for virus-induced gene silencing (VIGS) efficiency [31]. Phenotypes were scored at 15 dpi. Each silencing experiment was repeated four times, and each experiment included three independent plants.

### 4.7. Western Blot Analysis

Total protein was extracted from *N. benthamiana* leaf discs using Laemmli buffer at 2 dpi [32]. The proteins were then resolved by 12% SDS-PAGE and subjected to immunoblot analysis. Anti-GFP was used to verify the expression of SDE1mp-YC, while anti-Myc was used to verify the expression of NbDDX3-YN fusion.

### 4.8. Sequence Analysis

A BLAST search was performed in the Sol Genomics Network (https://solgenomics.net/) to identify *N. benthamiana* genes based on the cDNA sequence located in pGADT7. Conserved domains were analyzed by the NCBI Conserved Domain Search Tool.

## Supplementary Materials

Supplementary Materials can be found at https://www.mdpi.com/1422-0067/21/21/7996/s1. Figure S1, Schematic diagram for full length SDE1 and mature SDE1 (SDE1mp) for transient expression. The N-terminal 24 amino acids were deleted to generate SDE1mp; Figure S2, Alignment of amino acid sequences between NbDDX3 and CsDDX3; Figure S3, Silencing of *NbPSMD14* gene in *N. benthamiana*. Growth phenotypes were scored at 15 dpi. Death leaf in silenced plant is indicated by white arrows; Table S1, Bacterial strains and plasmids used in this study; Table S2, Primers for molecular cloning and qRT-PCR in this study.
