# Supplementary material for: ‘Candidatus Liberibacter Asiaticus’ SDE1 Effector Induces Huanglongbing Chlorosis by Downregulating Host DDX3 Gene
Source: Int J Mol Sci. 2020 Oct 27;21(21):7996. doi: 10.3390/ijms21217996 (PMC7662370; doi:10.3390/ijms21217996)
Supplement: Supplementary file 1 [file ijms-21-07996-s001.pdf]

Table S1. Bacterial strains and plasmids used in this study.

Table S1. Bacterial strains and plasmids used in this study

Strains or plasmids	Relevant characteristics	Resources
**Strains**		
Escherichia coli		
DH5α	*F^- recA hsdR17 (rk^-, mk^+) ϕ80lacZΔM15*	Clontech
Yeast		
AH109	MATa, *trp1-901, leu2-3, 112, ura3-52, His3-200, gal4, gal80, LYS2::GAL1UAS-GAL1TATA-His3*	Clontech
Agrobacterium tumefaciens		
GV3101	Rif^r, with Ti plasmid pMP90	[1]
**Plasmids**		
pHB	Km^r, a binary vector to express gene under control of a double CaMV 35S promoter	[2]
pHB-SDE1	Km^r, the 465-bp coding sequence of full length *SDE1* cloned in pHB	This study
pHB-SDE1mp	Km^r, the 393-bp DNA fragment coding for SDE1mp lacking signal peptide cloned in pHB	This study
pGDR	Km^r, RFP transient expression vector	[3]
pGDR-SDE1	Km^r, a 465-bp coding sequence of *SDE1* cloned into pGDR	This study
pGDR-SDE1mp	Km^r, a 393-bp coding sequence of SDE1mp cloned into pGDR	This study
pGDG	Km^r, GFP transient expression vector	[3]
pGDG-NbDDX3	Km^r, a 1413-bp *NbDDX3* gene cloned into pGDG	This study
pGBKT7	Km^r, GAL4(1–147) DNA-BD, TRP1, c-Myc epitope tag	Clontech
pGBKT7-SDE1	A 465-bp coding sequence of *SDE1* cloned in pGBKT7	This study
pGADT-7	Amp^r, *GAL4*(768-881) AD, *LEU2*, HA epitope tag	Clontech
pGADT7-CsDDX3	Amp^r , the 1413-bp *CsDDX3* genes cloned in pGADT7	This study
TRV1	Km^r, a VIGS vector encoding the replication, movement and cysteine-rich protein protoeins of tobacco rattle virus	[4]
TRV2	Km^r, a VIGS vector harboring the coat protein and two non-structural proteins of tobacco rattle virus	[4]

TRV-NbPDS	Km^r, a 369-bp DNA fragment of *NbPDS* cloned in TRV-RNA2	[4]
TRV-NbDDX3	Km^r, a 437-bp DNA fragment of *NbDDX3* cloned in TRV-RNA2	This study
1301-YC	Km^r, derivated from pCAMBIA1301 which encodes the C-terminal portion of YFP	[5]
YC-SDE1mp	Km^r, a 393-bp DNA fragment coding for SDE1mp cloned in 1301-Yc	This study
1301-YN	Km^r, derivated from pCAMBIA1301 which encodes the N-terminal portion of YFP	[5]
YN-NbDDX3	Km^r, a 1413-bp *NbDDX3* gene cloned in 1301-YN	This study
SRC2-1-GFP	Gm^r, a SRC2-1 GFP fusion in gateway vector pDONR207	[6]
pGADT7-VemR	Amp^r , the 384-bp full length of *vemR* genes cloned in pGADT7-AD vector at *Nde*I and *Eco*RI sites	[7]
pGBKT7-RpoN2	Km^r, the 1404-bp full length of *rpoN2* gene cloned in pGBKT7-BD vector at *Nde*I and *Eco*RI sites	[7]

References

1. Koncz, C.; Schell, J. The promoter of TL-DNA gene 5 controls the tissuespecific expression of chimeric genes carried by a novel type of *Agrobacterium* binary vector. *Mol. Gen. Genet*. **1986,** *204,* 383–396.
2. Mao, J.; Zhang, Y.C.; Sang, Y.; Li, Q.H.; Yang, H.Q. A role for *Arabidopsis* cryptochromes and COP1 in the regulation of stomatal opening. *Proc. Natl. Acad. Sci. USA* **2005,** *102,* 12270–12275.
3. Goodin, M.M.; Dietzgen, R.G.; Schichnes, D.; Ruzin, S.; Jackson, A.O. pGD vectors: versatile tools for the expression of green and red fluorescent protein fusions in agroinfiltrated plant leaves. *Plant J.* **2002,** *31,* 375–383.
4. Liu, Y.; Schiff, M.; Dinesh-Kumar, S.P. Tobacco *Rar1*, *EDS1* and *NPR1*/*NIM1* like genes are required for N-mediated resistance to tobacco mosaic virus. *Plant J.* **2002,** *30,* 415-429.
5. Li, Y.R.; Che, Y.Z.; Zou, H.S.; Cui, Y.P.; Guo, W.; Zou, L.F.; Biddle, E.M.; Yang, C.H.; Chen, G.Y. Hpa2 required by HrpF to translocate *Xanthomonas oryzae* transcriptional activator-like effectors into rice for pathogenicity. *Appl Environ Microbiol.* **2011,** *77*, 3809-3818.
6. Liu, Z.Q.; Qiu, A.L.; Shi, L.P.; Cai, J.S.; Huang, X.Y.; Yang, S.; Wang, B.; Shen, L.; Huang, M.K.; Mou, S.L.; Ma, X.L.; Liu, Y.Y.; Lin, L.; Wen, J.Y.; Tang, Q.; Shi, W.; Guan, D.Y.; Lai, Y.; He, S.L. SRC2-1 is required in PcINF1-induced pepper immunity by acting as an interacting partner of PcINF1. *J. Exp. Bot.* **2015,** *66,* 3683-3698.
7. Wu, W.; Zhao, Z.; Luo, X.; Fan, X.; Zhuo, T.; Hu, X.; Liu, J.; Zou, H. Response regulator VemR regulates the transcription of flagellar rod gene *flgG* by interacting with σ54 factor RpoN2 in *Xanthomonas citri* ssp. *citri*. *Mol. Plant Pathol.* **2019,** *20,* 372–381.

Table S2. Primers for molecular cloning and qRT-PCR in this study

**Primer pair**	**Sequence(5′-3′)**	**Cutting sites**	**Description or purpose**
Primers used for molecular cloning			
SDE1.pHB.F/SDE1.pHB.R	TTGGATCCATGCGTAAAAATTTATTAACCT<br>TTGAGCTCTCAAGACTGCTCCAACATTT	*Bam*HI-*Sac*I	A 465-bp coding sequence of full length *SDE1* cloned into pHB
SDE1mp.pHB.F1	TTGGATCCATGGGCAGTAGTTTTGGTTGTTG<br>(Combined with SDE1.pHB.R)	*Bam*HI-*Sac*I	A 393-bp DNA fragment coding for SDE1mp cloned into pHB
SDE1.RFP.F/ SDE1.RFP.R	TCGTCGACATGCGTAAAAATTTATTAACCT<br>TCGGATCCAGACTGCTCCAACATTTTTC	*Sal*I-*Bam*HI	A 465-bp coding sequence of *SDE1* cloned into pGDR
SDE1mp.RFP.F	TCGTCGACATGGGCAGTAGTTTTGGTTGTTG<br>(Combined with SDE1.RFP.R)	*Sal*I-*Bam*HI	A 393-bp coding sequence of *SDE1mp* cloned into pGDR
SDE1.BD.F/ SDE1.BD.R	TCGAATTCATGCGTAAAAATTTATTAACCT<br>TCCTGCAGAGACTGCTCCAACATTTTTC	*Eco*RI-*Pst*I	A 465-bp coding sequence of *SDE1* cloned into pGBKT7
NbDDX3.Ri.F/ NbDDX3.Ri.R	GCTCTAGAGCCTGGCTGGGTAAAG<br>GGGGTACCCAGAGCGATGAACAAAAGT	*Xba*I-*Kpn*I	A 437-bp DNA fragment of *NbDDX3* cloned in TRV-RNA2 for antisense
NbPSMD14.Ri.F/<br>NbPSMD14.Ri.R	GCTCTAGACCCTCCTACTCTGGACTCTT<br>GGGGTACCTCTACCGTGGCTCTTGG	*Xba*I-*Kpn*I	A 467-bp DNA fragment of *NbPSMD14* cloned in TRV-RNA2 for antisense
Niben101Scf05290g02006.1.Ri.F<br>/<br>Niben101Scf05290g02006.1.Ri.<br>R	GCTCTAGACAAAGCGGAGCAAACA<br>GGGGTACCCCACAAGATGAGGCAGAG	*Xba*I-*Kpn*I	A 438-bp DNA fragment of *Niben101Scf05290g02006.1* cloned in TRV-RNA2 for antisense
Niben101Scf04231g02014.1.Ri.F<br>/<br>Niben101Scf04231g02014.1.Ri.<br>R	GCTCTAGATAGGGAAACGAGTTGTGAA<br>GGGGTACCAGGGCGGAAGAAGGTC	*Xba*I-*Kpn*I	A 317-bp DNA fragment of *Niben101Scf04231g02014.1* cloned in TRV-RNA2 for antisense
NbDDX3.GFP.F/<br>NbDDX3.GFP.R	GCGTCGACATGCTCGCAGTTGGATTTG<br>CGGGATCCATAGTCCCTTTTGTTAGGGCA	*Sal*I-*Bam*HI	A 1413-bp *NbDDX3* gene cloned into pGDG

SDE1mp.Yc.F/ SDE1mp.Yc.R	GCTCTAGAATGGGCAGTAGTTTTGGTTGTTG<br>GGGGTACCAGACTGCTCCAACATTTTTC	*Xba*I-*Kpn*I	A 393-bp DNA fragment coding for SDE1mp cloned into 1301-YC
NbDDX3.Yn.F/ NbDDX3.Yn.R	GCTCTAGAATGCTCGCAGTTGGATTTG<br>GGGGTACCATAGTCCCTTTTGTTAGGGCA	*Xba*I-*Kpn*I	A 1413-bp *NbDDX3* gene cloned into 1301-YN
CsDDX3.F/CsDDX3.R	TCGAATTCATGCTTGCAGTTGGATTT<br>TCCTGCAGCAGTTAGGACATTCAGAT	*Eco*RI-*Pst*I	A 1488-bp *CsDDX3* gene cloned into pGADT7

Primers used for qRT-PCR analysis

**Gene**	**Sequence(5′-3′)**	**Description**
NbEF1a	TGGTGTCCTCAAGCCTGGTATGGTTG;<br>ACGCTTGAGATCCTTAACCGCAACATTCTT	160-bp
SDE1	CCATATGCGTCCTTTCACCAA; GAGATTGAGGAGCAGAACCCC	125-bp
NbDDX3	GTTTTCACACAGACAAAGCGAGATG; CCCTCTGATGTTGAGAAATGTCG	103-bp
CsCOX	GTATGCCACGTCGCATTCCAGA; GCCAAAACTGCTAAGGGCATTC	68-bp
CsDDX3	AAAACCTTCCACCCAAGCGACAGAG; CCCTCTGCAAGCTTTTCGTCTTGGT	139-bp

Underlined are restriction enzyme sites at 5′ end of each primers

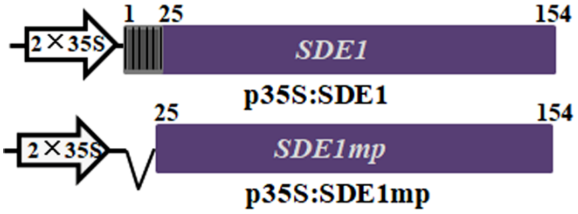

Figure S1 Schematic diagram of full-length SDE1 and mature SDE1 (SDE1mp) for transient expression. The N-terminal 24 amino acids were deleted to generate SDE1mp.

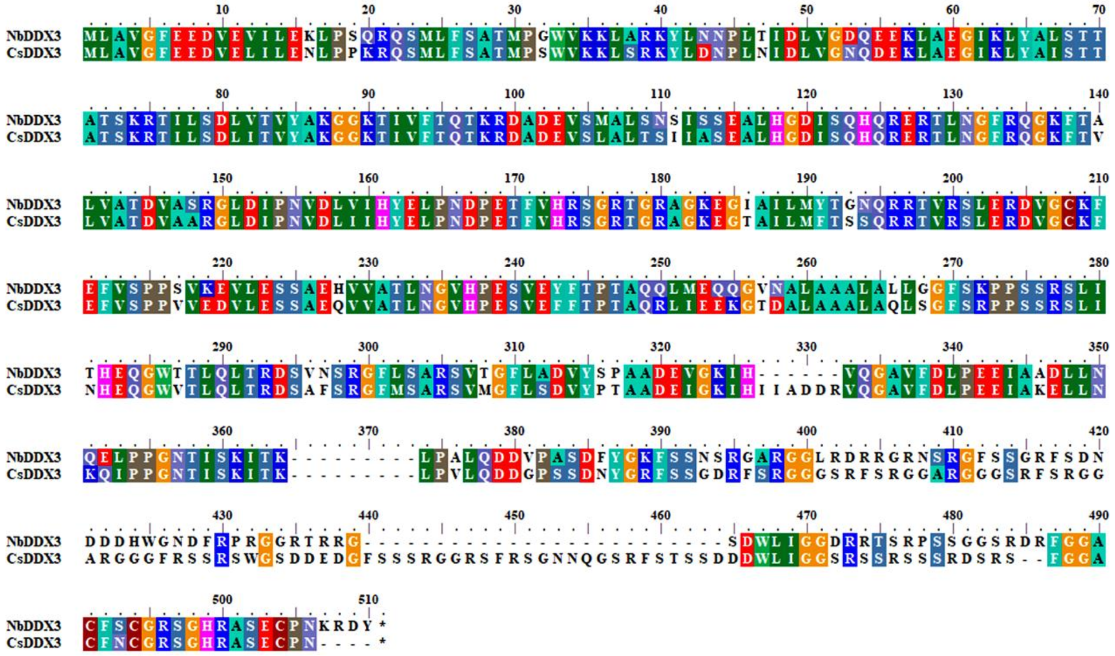

Figure S2 Alignment of amino acid sequences between NbDDX3 and CsDDX3.

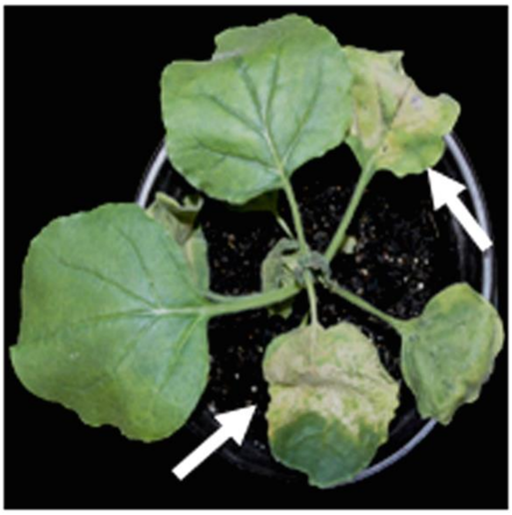

TRV:*NbPSMD14*

Figure S3 Silencing of *NbPSMD14* gene in *N. benthamiana*. Growth phenotypes were scored at 15 dpi. White arrows indicate leaf death in silenced plants.
